# Sugar feeding patterns of New York *Aedes albopictus* mosquitoes are affected by saturation deficit, flowers, and host seeking

**DOI:** 10.1371/journal.pntd.0008244

**Published:** 2020-10-26

**Authors:** Kara Fikrig, Sonile Peck, Peter Deckerman, Sharon Dang, Kimberly St Fleur, Henry Goldsmith, Sophia Qu, Hannah Rosenthal, Laura C. Harrington

**Affiliations:** Entomology Department, Cornell University, Ithaca, New York, United States of America; Faculty of Science, Mahidol University, THAILAND

## Abstract

**Background:**

Sugar feeding is an important behavior which may determine vector potential of female mosquitoes. Sugar meals can reduce blood feeding frequency, enhance survival, and decrease fecundity, as well as provide energetic reserves to fuel energy intensive behaviors such as mating and host seeking. Sugar feeding behavior can be harnessed for vector control (e.g. attractive toxic sugar baits). Few studies have addressed sugar feeding of *Aedes albopictus*, a vector of arboviruses of public health importance, including dengue and Zika viruses. To address this knowledge gap, we assessed sugar feeding patterns of *Ae*. *albopictus* for the first time in its invasive northeastern USA range.

**Methodology/Principal findings:**

Using the cold anthrone fructose assay with robust sample sizes, we demonstrated that a large percentage of both male (49.6%) and female (41.8%) *Ae*. *albopictus* fed on plant or homopteran derived sugar sources within 24 hrs prior to capture. Our results suggest that sugar feeding behavior increases when environmental conditions are dry (high saturation deficit) and may vary by behavioral status (host seeking vs. resting). Furthermore, mosquitoes collected on properties with flowers (>3 blooms) had higher fructose concentrations compared to those collected from properties with few to no flowers (0–3).

**Conclusions/Significance:**

Our results provide the first evidence of *Ae*. *albopictus* sugar feeding behavior in the Northeastern US and reveal relatively high rates of sugar feeding. These results suggest the potential success for regional deployment of toxic sugar baits. In addition, we demonstrate the impact of several environmental and mosquito parameters (saturation deficit, presence of flowers, host seeking status, and sex) on sugar feeding. Placing sugar feeding behavior in the context of these environmental and mosquito parameters provides further insight into spatiotemporal dynamics of feeding behavior for *Ae*. *albopictus*, and in turn, provides information for evidence-based control decisions.

## Author summary

Sugar feeding on plant nectar and other sources is an important mosquito behavior that varies between mosquito types. It is critical to understand sugar feeding because it impacts other aspects of mosquito biology, such as egg production, survival, and energy for activities such as mating and host seeking. Sugar can also be used to trap and kill mosquitoes. For example, attractive toxic sugar baits have been tested as a new control technique that depends on sugar feeding behavior for success. We investigated this behavior for the Asian tiger mosquito, a globally invasive species that can transmit several pathogens. We know very little about its sugar feeding behavior–only 4 studies have been conducted on the topic prior to ours, and none in Northeastern US, where our study was conducted. We found that hot and dry weather leads the mosquito to sugar feed more often and the presence of flowers increases the amount of sugar contained in those mosquitoes. Unexpectedly, we observed that host-seeking mosquitoes were more likely to be sugar fed than resting mosquitoes, which is contrary to previous studies showing a reduction in blood feeding after sugar feeding. In order to fully understand the patterns that we observed, further research will be necessary.

## Introduction

*Aedes albopictus* is a vector of numerous pathogens, including dengue, chikungunya and Zika viruses as well as dog heartworm parasites [1–3]. Its global range is rapidly expanding and pushing northward in the USA, enabled by local adaptation and winter egg diapause [4, 5]. This highly adaptable mosquito can survive in drastically varied ecosystems, ranging from tropical to temperate climates, making it one of the most successful invasive species globally [6]. Understanding this mosquito’s feeding behavior and ecology across its invasive range is essential for understanding risk and devising control methods.

Sugar feeding is an important mosquito behavior with implications for disease transmission and control [7]. It can impact mosquito life history through a number of mechanisms and can vary between mosquito species [8]. For females, there may be trade-offs in transmission potential between blood and sugar feeding [8], as the latter may lead to satiation and reduce available abdominal space for a blood meal shortly after sugar feeding [9]. Sugar feeding has been considered a blood feeding suppressant in *Ae*. *albopictus* and other mosquito species, reducing blood meal size and frequency, thereby reducing opportunities for pathogen transmission [8, 10, 11]. In contrast, sugar feeding can enhance survival of *Ae*. *albopictus* and other mosquito species in laboratory studies, potentially increasing pathogen transmission [8, 10, 12–17]. Sugar also may enhance male mosquito mating performance by providing energy for mate-seeking and swarming [13, 17–20] and enable female host-seeking behavior [20]. In addition to impacts on mosquito life history, sugar feeding behavior has implications for the success of certain control and surveillance methods, such as attractive toxic (or targeted) sugar baits (ATSBs), which contain sugar and flower-derived attractants mixed with insecticides [21].

Environmental drivers that vary widely across *Ae*. *albopictus* invasive range can influence feeding behavior. For example, fructose feeding rates can vary by season and location, which may be caused by differences in temperature and humidity [22–26]. Dehydration due to low humidity conditions may stimulate sugar feeding behavior as has been shown by Hagan et al. (2018) for blood feeding [27]. Availability of sugar sources such as floral nectar may also affect mosquito sugar feeding rates, especially in arid climates [24, 28–30]. However, in addition to flower nectar, mosquitoes can acquire sugar from plant leaves, fruit, and homopteran honeydew [15, 24, 25, 31, 32] and these alternative sources can vary across *Ae*. *albopictus* habitats.

Given the importance of sugar feeding for mosquito fitness and the public health threat of *Ae*. *albopictus*, we know surprisingly little about its sugar feeding patterns in nature. Only four field studies have been conducted across vastly different habitats [17, 24, 33, 34]. Two studies indicated that season, habitat, and sugar availability might be important, as well as temperature and humidity [17, 24].

In Israel, the percent of sugar positive *Ae*. *albopictus* varied by season and habitat type (irrigated garden versus dry wasteland), ranging from 41.4% (summer) to 74.1% (fall) at a wasteland site [24]. However, this study was performed with an abbreviated cold anthrone assay, using visual detection of color change, instead of precise measurement of fructose concentration using established methods [24]. A subsequent study evaluating *Ae*. *albopictus* visitation to sugar sources reported attraction to a subset of tested ornamental flowers, wildflowers, damaged carob seed pods and fruits, but no attraction was detected to honeydew coated plants [34]. Working with releases of laboratory colony males (F_33_-F_47_) in northern Italy, Bellini et al. (2014) utilized an abbreviated cold anthrone assay to detect higher sugar feeding rates for released males at sites with sucrose feeding stations compared to control sites and a positive correlation with temperature and negative correlation with humidity [17]. In Florida, where the only other US study was conducted, *Ae*. *albopictus* fructose concentration did not vary significantly with plant species utilized as resting habitat; unfortunately, no analyses were conducted to determine the proportion sugar fed [33]. Another limitation of these studies was the lack of established baseline fructose levels, leading to the potential misidentification of larval nutrients as adult sugar meals.

The current knowledge of *Ae*. *albopictus* sugar feeding in the field primarily stems from these four studies in Israel, Italy, and Florida. Additionally, assessments of ATSBs for *Ae*. *albopictus* population control in Florida and Israel have demonstrated that sugar feeding frequency is sufficient to achieve population reductions [35–39]. However, these locations are not representative of the vast environmental variation in climate and flora where *Ae*. *albopictus* is now established. It has yet to be determined whether sugar feeding behavior of *Ae*. *albopictus* in other regions of the world will be conducive to ATSB success due to an absence of basic ecological and behavioral information on this subject.

To address this important gap, we assessed the sugar feeding behavior of *Ae*. *albopictus* at its invasive edge in Northeastern USA in order to understand its feeding ecology along the northern limit of its expanding range. We determined the proportion of male and female mosquitoes that contained fructose and individual mosquito fructose concentrations. In addition, we assessed the response of sugar feeding to saturation deficit (environmental dryness), floral presence, host seeking status, and sex. Placing sugar feeding behavior in the context of these environmental and mosquito parameters provides further insight into spatiotemporal dynamics of this behavior for *Ae*. *albopictus*, and in turn, provides information for evidence-based control decisions.

## Methods

### Field site

**Fig 1:**
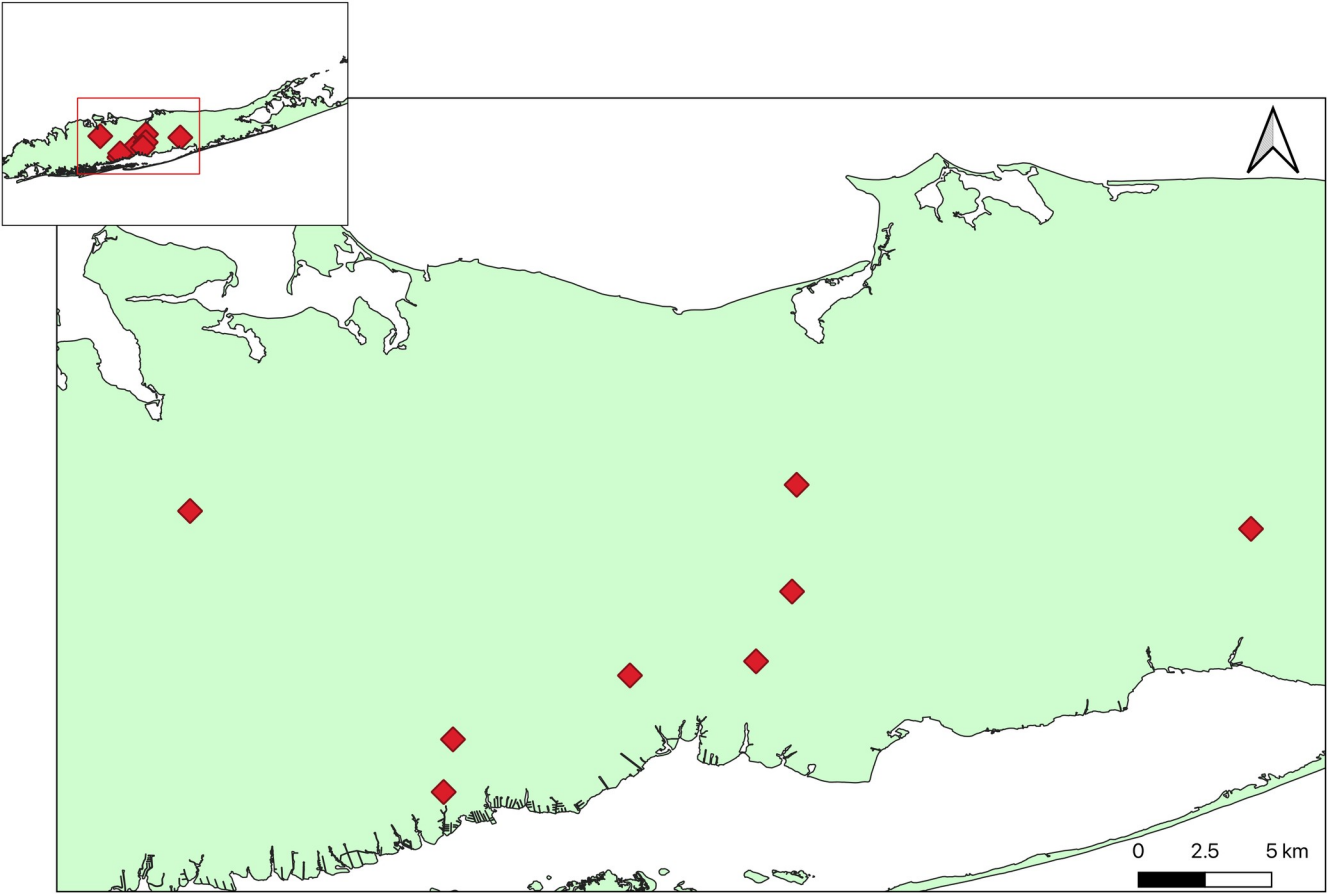
Map of site locations on Long Island, New York. Red diamonds indicate site locations. Small box in the upper left shows zoomed out map of entire island.

Mosquitoes were collected in Long Island, New York, USA, at four farms and four residential areas with 9–17 houses in each, totaling 50 properties. Sites were chosen based on prior knowledge of *Ae*. *albopictus* distribution in Suffolk County from larval surveys and vector control surveillance [40] (S. Campbell, pers comm). The eight sites were located in separate towns spanning a substantial section of Long Island (40km East to West and 15km North to South) (Fig 1). All four farms were surrounded to some degree by both forested and residential land. The four residential areas had variable levels of vegetation, both within and between sites. Residential property sizes ranged from approximately 200 – 1200m^2^. Terrain was flat across all 8 sites (elevation range approximately 3–79m above sea level). Collections were conducted between June and August 2018. Two HOBO Pro v2 data loggers (model U23-001, Onset Computer Corp., Bourne MA, USA) per site recorded the temperature and humidity every four hours from mid-July through August.

### Mosquitoes

Resting mosquitoes were collected using large custom-designed aspirators (30.5 cm diameter, 114 cm height, 12 V PM DC 2350 RPM, 1/35 Horse power, 3.7 Amp motor) [41] and host seeking mosquitoes that approached collectors were caught with nets. All properties at the eight sites were sampled once per week between 8:00 and 19:00 hrs. The only exception was a small number of individual properties (n = 11) where permission to collect was not provided on some weeks. Two collection teams of three people worked simultaneously at separate properties in residential areas and together at farms. Aspirator collections were conducted by two individuals per property for the length of time necessary to thoroughly sample the entire property, which varied with size and complexity of landscape (most aspirator collection times were between 7-12min; range from 2.5-17min). Host seeking collections were not initially planned, but were included after large numbers of host seeking mosquitoes were observed during initial collection days. The host seeking collections were therefore conducted opportunistically by a third person responsible for specimen labeling and by all three collectors while sorting through aspirator collections after bags were placed in acetone jars for ~3 min. Anesthetized mosquitoes were then separated into microcentrifuge tubes, placed on ice and transported to the laboratory. Mosquitoes were identified to species using published keys [42], sorted by blood meal status, labeled, and stored at -20°C. A small number of the blood-fed mosquitoes (182) were saved for later blood meal analysis and non-blood fed mosquitoes were utilized for sugar analysis. Mosquitoes were transported on dry ice to Cornell University for further processing. To determine body size, one wing was removed from each mosquito, placed on a slide and measured from the axillary incision to base of fringe hairs [43] using a dissecting microscope and software (Olympus SZX9, Olympus DP22 camera, and Olympus cellSens software).

### Flower census

Beginning in mid-July 2018, the number of blooming flowers per morphospecies (up to 100 blooms) was counted on each farm and residential property (n = 54). Morphospecies (morphologically distinct species) were identified using the GardenAnswers phone application (Garden Answers LLC., San Diego, CA) [44]. Flower species varied between properties, including both ornamental and wildflowers consisting of a wide spectrum of different colors, shapes and sizes. However, species-level identifications were not verified by experts and were therefore not analyzed further. Flowers were categorized into groups representing flower presence: absent (0–3 blooms) and present (>3 blooms). A range of zero to three was chosen to represent an absence of flowers rather than zero because three was a natural break point in the data, with only one mosquito collected on a property with 6 or 8 flowers, and all others on properties with at least 9 flowers, creating a natural gap between mosquitoes collected on properties with 0–3 blooms and the rest of the dataset. Properties with up to three flowers had relatively little nectar and were therefore considered an appropriate comparison group to more highly flowered properties, expanding the number of mosquito observations on ‘absent’ properties by 50% compared to an absolute zero ‘absent’ group.

### Fructose detection

#### Cold anthrone assay

Fructose concentration was measured using the cold anthrone colorimetric assay [45]. At room temperature, anthrone solution reacts with fructose, but not other sugars. The assay is indicative of plant feeding and does not measure blood sugars (primarily glucose) or stored sugars (trehalose), although non-sugar fed teneral mosquitoes contain small amounts of fructose.

Mosquitoes were homogenized in 1.7 ml microcentrifuge tubes using a lyser (FastPrep-24 Classic Instrument, MP Biomedicals, USA) at 4 m/s for 30 s with 50 μl of 2% sodium sulfate solution and glass beads (3 mm, Thermofisher). Chloroform methanol (1:2) solution (375 μl) was added to each tube and vortexed for 8 s and centrifuged for 15 min at 200 x g, extracting fructose into supernatant. Tubes were stored at -20°C until fructose quantification, at which time 10 μl of supernatant was transferred to two wells of a 96-well microplate.

To ensure consistency, standards were produced once via serial dilution and stored at 4°C for the duration of analysis. Two replicates of standards (10 μl of 0, 0.05, 0.1 and 0.2 μg /μl D-Fructose (Fisher Chemical, USA) in 25% ethanol) and samples (10 μl) were pipetted individually into wells on each plate. Thereafter, 240 μl anthrone solution (freshly prepared each day, 67.9 μl distilled water, 172.1 μl sulfuric acid, and 0.339 mg anthrone per sample) was added with a multichannel pipette. Samples were incubated at room temperature for 90 min in a chemical hood. The absorbance of light (630 nm) by the reaction of each sample was measured by the microplate reader (800 TS Absorbance Reader, BioTek, VT, USA) and compared against the standard curve to determine fructose concentration. The mean of the two experimental replicates of each sample was used in analyses, except when experimental replicates were dissimilar, the data were discarded or the sample was reanalyzed.

#### Baseline mosquito fructose concentrations

During the period of adult collection, pupae were collected from containers on a subset of properties and held in the laboratory until eclosion. Post-eclosion, adult *Ae*. *albopictus* (n = 78 male, 53 female) were held without sugar and frozen within 12 hrs of emergence followed by sugar analysis with the cold anthrone assay. One male and one female outlier were removed using the Median Absolute Deviation. The remaining mosquito data were used to establish a field baseline level of fructose in teneral *Ae*. *albopictus* [22]. Field-collected adults with fructose concentrations greater than one standard deviation above the sex-specific mean baseline concentration were considered to be fructose-positive.

#### Laboratory digestion assay

To determine the time window of fructose detection in *Ae*. *albopictus* post-sugar meal consumption, we conducted an assay of fructose concentration in sugar fed females over digestion time [46]. *Aedes albopictus* (F6 from NY at 23.5°C and F8 from FL at 28°C) were vacuum hatched and provided with a pinch of pulverized fish food (crushed Cichlid Gold fish food pellets; Hikari, Himeji, Japan). One day later, they were separated into trays of 200 larvae with 1L of distilled water and 4 Cichlid Gold fish food pellets. Pupae were transferred to cages and fed 10% sucrose solution between 1–3 d post-eclosion. Males and females were removed before, immediately after, and at 24 hr intervals post sugar feeding. Between nine and twenty mosquitoes were removed per day. Fructose concentration was measured as described above. The assumption of constant variance was not met, so mean fructose concentrations were compared to concentration before feeding using the non-parametric Kruskal-Wallis test followed by a Dunn’s multiple comparisons test with Benjamini-Hochberg correction (reported as P_adj_).

### Data analysis of sugar feeding patterns in the field

Analyses were performed in R (Version 1.1.463) [47]. Average fructose concentration and proportion sugar fed were calculated for all male and female *Ae*. *albopictus* collected from June to August 2018. Wing measurements were used to standardize fructose concentration by body size, by dividing total concentration by mm wing length. A subset of *Ae*. *albopictus* for which we had flower and weather data (those collected between 23 July—15 August) were included in the models described hereafter.

A Generalized Linear Mixed Model (GLMM; lme4 package) with binomial distribution was employed to determine the impact of measured variables on sugar feeding probability of a captured mosquito [48]. Random effects included town, address nested in town, date, town-date interaction, and address nested in town-date. Fixed effects included capture method (aspirator or net), sex, presence of open flower blooms on property, and saturation deficit. Initially, several weather parameters were evaluated, including minimum, maximum, and average temperature and humidity, as well as saturation deficit [49].

$$SD=(1-\frac{RH}{100})4.9463e^{0.0621T}$$

All weather parameters had similar explanatory power in the models, so saturation deficit was chosen for the final model because it included both temperature and humidity in a biologically relevant way. Because the sugar was detectable for up to 24 hrs after consumption in our laboratory assessments, the cumulative saturation deficit over that time was determined by summing the saturation deficit over the six most recent time points (a 24 hr interval) prior to collection time for each mosquito. Flower count was included as a binary variable measuring flower presence as described above.

A linear mixed model was employed to evaluate log fructose concentration standardized by wing length using all the fixed and random effects listed above. Only mosquitoes that were sugar fed were included in this analysis to further understand factors influencing the magnitude of sugar feeding.

For both GLMM and linear mixed models, post hoc analyses were conducted by calculating the estimated marginal means of the effects of individual parameters using the emmeans package [50].

## Results

### Environmental and flower measurements

For the dates July 23 –August 15, 2018, mean ± SD temperature was 24.4 ± 2.79°C (range 15.4°C—37.1°C). Mean relative humidity was 87.1 ± 12.2% (range 1%-100%). Floral counts varied by property visit (collection event on a given property): more properties had flowers present (154) during mosquito collections than absent (20). The median number of flowers per property was 110.5.

### Mosquitoes

Between June and August 2018, 2,788 *Ae*. *albopictus* were collected; 1,263 females (45.3%) and 1,525 males (54.7%). Of these, 2,517 (90.3%) were collected resting on vegetation and other surfaces by aspirator and 271 (9.7%) were captured flying around human collectors with nets (241 female and 30 male). Mosquitoes were captured across 8 sites, with 1,097 (39.3%) from the four farms and 1,691 (60.7%) from the four residential areas. Among the subset of mosquitoes that were captured during the floral census (1,970), 1,827 (92.7%) were collected on properties with flowers present and 143 (7.26%) on properties with flowers absent.

Female wings were 2.71 ± 0.27mm (mean ±SD; range: 1.59–3.50mm) and male wings were 2.22 ± 0.22mm (range 1.24–3.21mm).

### Fructose detection

#### Field-caught mosquitoes

Among mosquitoes collected from June through August, a high proportion of both male (756/1,525, 49.6%) and female (528/1,263, 41.8%) *Ae*. *albopictus* were sugar fed. The percent of sugar fed mosquitoes by each variable is displayed in Table 1. Among sugar fed mosquitoes, average female fructose concentration was 0.0488 μg/μl and male fructose concentration was 0.0300 μg/μl. To account for differences in body size, fructose concentrations were standardized by wing length for sugar fed females (0.0180 ± 0.0182 μg/(μl*mm)) and males (0.0134 ± 0.0132 μg/(μl*mm)). Average total fructose content was 18.3μg for females and 11.25μg for males.

**Table 1:** Sugar fed status of female and male mosquitoes by site, host seeking status, and floral presence.

	Female			Male		
	N (%)			N (%)		
	Sugar	No sugar	Total	Sugar	No sugar	Total
**Site type**						
Farm	206 (38.4)	330 (61.6)	536	260 (46.3)	301 (53.6)	561
Residential	322 (44.3)	405 (55.7)	727	496 (51.5)	468 (48.5)	964
**Host Seeking Status**						
Resting	398 (38.9)	624 (61.1)	1,022	738 (49.4)	757 (50.6)	1,495
Host seeking	130 (53.9)	111 (46.1)	241	18 (60.0)	12 (40.0)	30
Flowers*						
Absent	17 (36.2)	30 (63.8)	47	50 (52.1)	46 (47.9)	96
Present	308 (38.5)	492 (61.5)	800	457 (44.5)	570 (55.5)	1,027

Statistical tests of these differences are discussed in GLMM and linear mixed model results below

*Flower presence was only assessed beginning on July 23, so count reflects number collected thereafter

#### Baseline fructose concentration

Mean ± SD fructose concentrations from field collected pupae that eclosed in the laboratory without sugar were 1.42 ± 2.76 ng/μl for females and 0.935 ± 2.09 ng/μl for males. Baseline concentrations (mean fructose concentration +1 SD) were 4.18 ng/μl for females and 3.02 ng/μl for males. All field-captured adult fructose values above this baseline level were considered sugar fed.

#### Laboratory digestion assay

**Fig 2:**
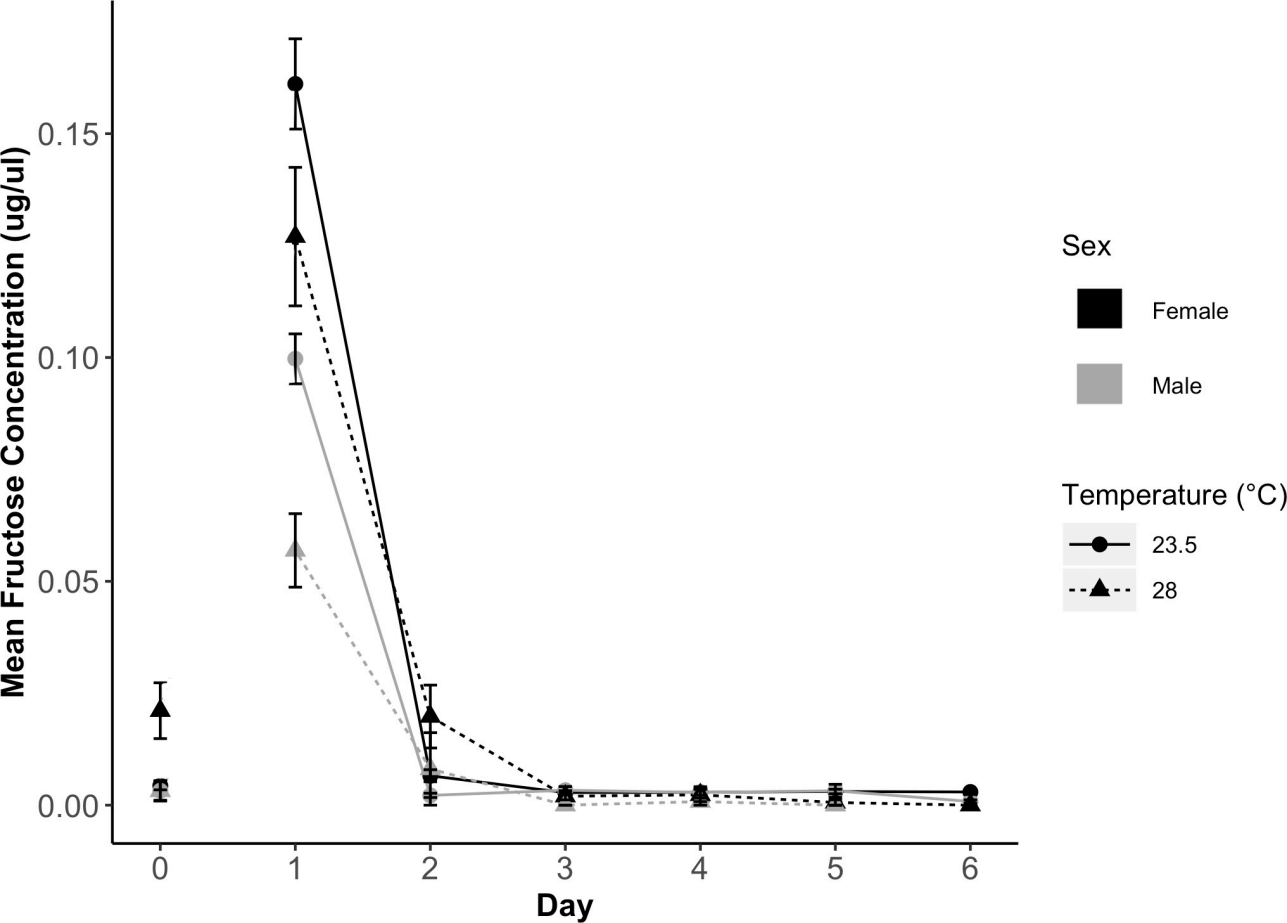
Male and female digestion of fructose over time at 23.5°C and 28°C. Fructose concentration was measured daily after time of ingestion (Day 1). The daily mean (±SE) fructose concentration is shown for females (black) and males (gray) at 23.5°C (solid line and circle points) and 28°C (dotted line and triangle points). Compared to pre-ingestion fructose concentrations (Day 0), mosquitoes only had significantly higher fructose concentrations on Day 1 (immediately after ingestion) for each sex at each temperature (Kruskal-Wallis Dunn’s test; P_adj_<0.05).

Male and female *Ae*. *albopictus* digested fructose within 24 hrs after ingestion (Fig 2) at both low (23.5°C) and high (28°C) constant temperatures. Compared to fructose levels before sugar feeding (Day 0), fructose was only detectable on Day 1, immediately after feeding (Kruskal-Wallis test with post-hoc Dunn’s test; female 23.5°C: P_adj_ = 0.0051; male 23.5°C: P_adj_ = 0.0004; female 28°C: P = 0.0099; male 28°C: P = 0.0001). At the next check point, 24 hrs post-feeding (Day 2), and all days thereafter (Days 3–6), fructose concentrations were either not different from (Kruskal-Wallis post-hoc Dunn’s, P_adj_>0.05) or lower (Female 28°C Day 5: P_adj_ = 0.0105 and Day 6: P_adj_ = 0.0052) than concentrations before sugar feeding.

### Adult *Ae*. *albopictus* sugar feeding patterns

#### Effects of environmental and mosquito parameters on sugar feeding status

**Fig 3:**
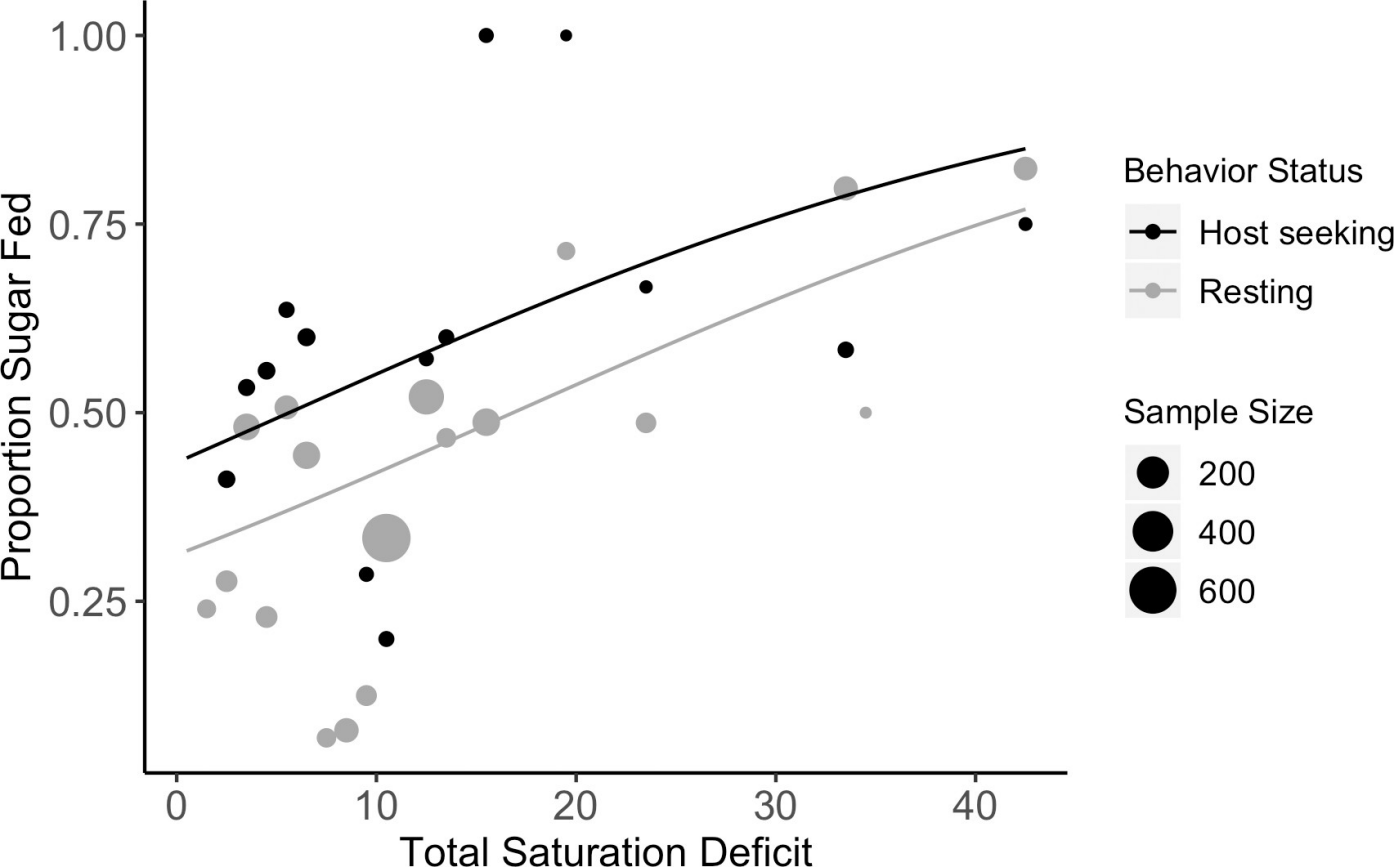
The proportion of sugar fed mosquitoes by saturation deficit for host seeking (black) and resting (gray) mosquitoes. Mosquitoes were grouped by 1 unit of saturation deficit. The total number of mosquitoes collected per unit saturation deficit is represented by point size. The predicted probability of sugar feeding by saturation deficit is indicated by the lines. As saturation deficit increased, the likelihood of capturing a sugar-fed mosquito increased (GLMM, *P* = 0.00143). Mosquitoes captured while host seeking were more likely to be sugar fed than while resting (GLMM, *P* = 0.00870) [50].

For the subset of mosquitoes captured after floral and weather data collection was initiated, saturation deficit, host seeking status, and sex influenced the probability of sugar feeding while the number of flowers on a property did not. The likelihood of sugar feeding was affected by dryness as measured by saturation deficit (n = 1,970 mosquitoes, β = 0.0470, SE = 0.0148, *P* = 0.00143). More mosquitoes fed on sugar when the saturation deficit was high (i.e. when weather was hotter and drier) (Fig 3). Host seeking mosquitoes (n = 151) were more likely to be sugar fed than resting individuals (n = 1,673; β = 0.527, SE = 0.201, *P* = 0.00870). Males (n = 1,042) were more likely to be sugar fed compared to females (n = 782; β = 0.394, SE = 0.110, *P* = 0.000321). The relative abundance of flowers did not affect the likelihood of sugar feeding; mosquitoes collected on properties with flowers (n = 1,827) were not more likely to be sugar fed than those captured on properties with no flowers (n = 143; β = 0.0728, SE = 0.290, *P* = 0.802).

#### Effects of environmental and mosquito parameters on fructose concentration ingested

**Fig 4:**
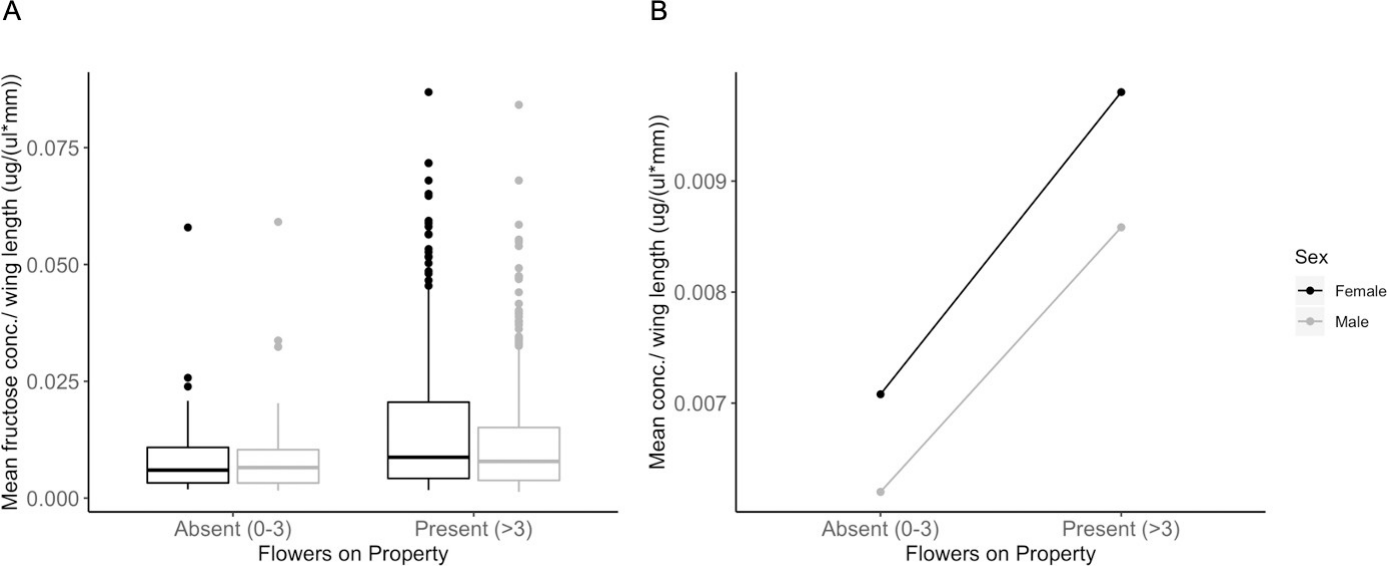
**A. Mean fructose concentration standardized by wing length for female (black) and male (gray) *Ae*. *albopictus* on properties with and without flowers.** Points show individual mosquito fructose concentration standardized by wing length of outliers. Includes both resting and host seeking mosquitoes. **B. Predicted fructose concentration by flower presence and sex**. Mosquitoes collected on properties with flowers present (LMM, *P* = 0.0253) had higher fructose concentration per mm wing length than those collected on properties with flowers absent. Females had marginally higher fructose concentration compared to males (LMM, *P* = 0.0553).

The linear mixed model results showed that the fructose concentration in sugar fed mosquitoes was predicted by flower abundance but not by saturation deficit, sex, or host seeking status. Among sugar fed mosquitoes (n = 832), those collected on properties with flowers present (n = 765) had significantly higher fructose concentration per mm wing length than those collected on properties with flowers absent (n = 67) (β = 0.325, SE = 0.142, *P* = 0.0253) (Fig 4). Males (n = 507) took marginally smaller sugar meals compared to females (n = 325) even when controlling for body size differences between the sexes (β = -0.133, SE = 0.0691, *P* = 0.0553). There was no significant effect of host seeking status (host seeking vs resting, β = 0.217, SE = 0.116, *P* = 0.0611) or saturation deficit (β = 0.00844, SE = 0.00711, *P* = 0.253) on fructose concentration per mm wing length.

## Discussion

Sugar feeding patterns of field captured *Ae*. *albopictus* mosquitoes have only been reported from three other locations: Israel, Italy, and Florida [17, 24, 33]. Our study reports Asian tiger mosquito sugar feeding patterns for the first time from the northern edge of its invasion in the Eastern USA. Using robust sample sizes, we demonstrated that a large proportion of both male and female *Ae*. *albopictus* fed on plant or homopteran derived sugar sources within 24 hrs of capture. Our results suggest that sugar feeding behavior increases when environmental conditions are dry and may vary by behavioral status (host seeking vs resting). Furthermore, mosquitoes collected on properties with flowers had higher fructose concentrations compared to those collected from properties with no flowers.

A large percentage of males (49.6%) and females (41.8%) collected from our field sites were sugar fed. Our laboratory assays demonstrated that *Ae*. *albopictus* digest fructose within 24 hrs of consuming a sugar meal at 23.5 and 28°C. According to this window of detection, and considering the average field temperatures during collections, approximately half of the field-captured mosquitoes fed on sugar daily. Sugar feeding estimates may be influenced by the concentration and composition of sugar consumed, which varies between flower species’ nectar and between alternative sources of sugar. In our study we used a 10%-sucrose solution representing the low end of sugar concentrations in nectar (7–70%) and only one of the constituent sugars, consistent with prior sugar digestion studies [46, 51]. Sucrose is a disaccharide, containing a glucose and fructose moiety (two other common nectar sugars); disaccharides are known to react in a similar manner to their monosaccharide constituents in the cold anthrone assay, reducing potential sources of variation [52]. However, it is possible that *Ae*. *albopictus* digestion rate of the sugar source we tested with laboratory mosquitoes may not be representative of all available natural sugar sources. In a temperate region of Italy, similar rates of sugar feeding were detected among released males (48% at 72 hours post-release) compared with wild males in our study [17]. In the arid climate of Israel, sugar feeding tended to be more common; the percentage of sugar fed mosquitoes ranged from 41.3% to 74.1% based on season and site [24].

In Long Island, *Ae*. *albopictus* that experienced higher saturation deficits (hotter, drier weather) during the 24 hours prior to collection were more likely to contain a sugar meal than those collected during lower saturation deficits. Bellini et al. (2014) observed a similar pattern with field-released males when assessing sugar feeding devices; the percentage of sugar positive males was correlated negatively with relative humidity and positively with temperature at control sites [17]. It is possible that high saturation deficit leads to dehydration and ultimately triggers higher rates of sugar feeding, especially on more dilute sources. Maintaining water balance is essential for insect survival [53, 54] and others have described insect foraging behaviors that balance physiological needs for water and sugar through choice of nectar dilution levels [55–58]. Working with mosquitoes, Hagan et al. (2018) found that blood feeding was prompted by dehydration [27]. Although sugar and blood feeding are different behaviors and dilute nectars can contain similar or lower levels of water compared to blood, it is possible that mosquitoes use the same set of physiological cues to prompt sugar feeding under dehydrating conditions. Upshur et al. (2019) demonstrated that sugar feeding increased between 20°C and 30°C, further suggesting the impact of environmental conditions on the tendency of mosquitoes to ingest sugar [59].

In our study, the presence of flowers did not influence the likelihood of *Ae*. *albopictus* sugar feeding but did impact the amount of sugar ingested when they did feed. Residential property sizes varied in our study but tended to be small and within the flight range of *Ae*. *albopictus* [60, 61], so it is possible that sugar fed mosquitoes collected in yards without flowers originally sugar fed in adjacent yards with greater floral abundance, and subsequently used some of the fructose in flight. This could explain why we observed consistent likelihood to sugar feed between flower categories, but different fructose concentrations between mosquitoes collected on properties with and without flowers. Alternatively, sugar fed mosquitoes in yards without flowers may have consumed non-nectar sources, such as honeydew or plant tissue. Parasitoid wasps fed on honeydew had lower fructose levels compared to those fed on nectar [62] and plant leaves generally have lower concentration of sugars than nectar [63]. This would also account for the equal likelihood of feeding and different fructose concentrations by flower presence.

Only one other published study has investigated floral abundance and *Ae*. *albopictus* sugar feeding and differs from our results. In Israel,under arid environmental conditions, Müller et al. (2010) found a difference in sugar feeding likelihood by flower abundance: 42% and 68% of females from low and high floral abundance sites, respectively, contained sugar [24]. It is difficult to compare the two studies due to substantial differences in environmental conditions. Houses without flowers in Long Island, USA still had significant vegetation and potential non-nectar sugar availability, in contrast to the less vegetated “dry wasteland” site in Israel. Sampling limitations in our study restricted floral surveys to properties where mosquitoes were collected, preventing inclusion of flowers in neighboring yards within the flight range of *Ae*. *albopictus*. Quality of floral resource was also not considered, such as nectar quantity or quality, which can be highly variable [64]. The design of the floral surveys also prevented analysis of floral density or species effects on sugar feeding.

A subset of host seeking mosquitoes were opportunistically captured with nets as they flew around human collectors. These mosquitoes were more likely to contain sugar meals than those collected with aspirators while resting on vegetation and other surfaces. While some studies have reported reduced blood feeding after sugar feeding [8, 10, 11], it is possible that teneral females seek sugar meals shortly after eclosion before blood feeding [9], explaining higher sugar content in host seeking females. This observation warrants further, more systematic investigation. In addition, it highlights the importance of considering collection method biases when assessing sugar feeding prevalence and should be an important consideration when designing and analyzing sugar feeding study results.

These sugar feeding patterns will likely influence the success of sugar-based control techniques, such as ATSBs. While this control strategy has only been assessed for *Ae*. *albopictus* populations in Florida and Israel [21, 35–39], our results provide insight into the potential for deployment of ATSBs in our study region. In Israel, 62.7% of female *Ae*. *albopictus* were sugar fed at a natural garden site; meanwhile, ATSB deployment reduced biting pressure by 85% at another site under similar conditions [24, 37]. The comparatively lower percentage of sugar fed females in Long Island (41.8%) may result in weaker reductions in biting pressure in our study region, but the sugar feeding rates were nevertheless sufficient to warrant further investigation of ATSB-based control methods in Northeastern US. Our results suggest that control success in our region may be maximized if ATSBs are deployed during hot, dry conditions and in locations with fewer flowers and less competition. Furthermore, the tendency of *Ae*. *albopictus* to sugar feed prior to blood feeding may increase the public health impact of ATSBs by concentrating control pressure before the point of pathogen acquisition or transmission.

While relatively little is known about *Ae*. *albopictus* sugar feeding, this behavior has been studied in other mosquito species, including *Ae*. *aegypti*, which shares some ecological similarities. In Thailand, the percentage of sugar fed *Ae*. *aegypti* females increased in the dry season (16%) compared to the rainy season (5%), potentially echoing the effect of saturation deficit on proclivity to feed found in our study [22]. Other studies have also shown remarkably low levels of sugar feeding for female *Ae*. *aegypti* [28, 65]. However, *Ae*. *aegypti* females in Texas had higher rates of sugar feeding (47.91%), similar to what we report for *Ae*. *albopictus* in our current study [66]. Another important vector species, *Anopheles gambiae*, had low rates of sugar feeding in Kenya; the percentage was higher for host seeking (14.4%) females compared to resting (6.3%), similar to the trend found in our study [67]. However, recent studies suggest that *An*. *gambiae* may feed on sugar more often than originally thought; populations can be successfully controlled by ATSBs [68], survival is reduced by removal of a flowering invasive shrub [30], and both males and females are robustly attracted to a number of different plants [69].

As the ability to detect DNA from mosquito plant meals improves, future studies could explore sugar feeding with greater resolution than the cold anthrone test affords. Next-generation sequencing has been employed to successfully identify plant meals of mosquitoes and other blood feeding Diptera [70, 71]. Additional studies of *Ae*. *albopictus* plant meal origin would be beneficial in ATSB lure design optimization. However, results of these analyses must be interpreted with caution as they may bias towards non-nectar sugar sources that are more likely to be detected via DNA-based analyses due to minimal DNA content of nectar.

Our results demonstrate, for the first time, sugar feeding patterns by temperate populations of *Ae*. *albopictus* in the United States. This is only the fourth field study on this important mosquito behavior and provides us with insights into conditions that might influence sugar feeding variation, including saturation deficit, flower presence, and host seeking. In light of the high frequency of sugar feeding in the study population, our results show promise for deployment of attractive toxic sugar baits for *Ae*. *albopictus* control in the region and provide insight into potential modifications of bait timing and placement to maximize success.
